# Supplementary material for: AvP: A software package for automatic phylogenetic detection of candidate horizontal gene transfers
Source: PLoS Comput Biol. 2022 Nov 9;18(11):e1010686. doi: 10.1371/journal.pcbi.1010686 (PMC9678320; doi:10.1371/journal.pcbi.1010686)
Supplement: S1 File — (PDF) [file pcbi.1010686.s001.pdf]

Comparing AvP results on *C. elegans* to Crisp et al., 2015

Genes defined as HGT in *C. elegans* by *Crisp et al., 2015* [1] : 139

- Annotated as pseudogenes in WormBase : 3
- Deleted from Wormbase: 1

Genes tested with AvP : 135

- No HGT: 21 [with hits from both metazoan and non metazoan: 17]
- HGT: 98 [with hits from both metazoan and non metazoan: 18]
- Complex: 16

Table S1: Manual Inspection of the trees of the 51 genes containing both metazoan and non metazoan hits (N=No HGT; H=HGT; C=Complex)

AvP result	Confirmed	$AI > 0$ $AHS > 0$	$AI < 0$ $AHS > 0$	$AI > 0$ $AHS < 0$	$AI < 0$ $AHS < 0$
	N		1	2	5
N=17	H	1*	4*		
	C		4†		
C=16	C	6	1	8	1
H=18	H	16	1		
	C			1§	

* db error, nematode protein classified as non nematode metazoan
† possible independent hgt in another metazoan
§ db error, nematode protein classified as bacteria

After manual inspection

- No HGT: 12 [with hits from both metazoan and non metazoan: 8]
- HGT: 102 [with hits from both metazoan and non metazoan: 22]
- Complex: 21

References

[1] Crisp A, Boschetti C, Perry M, Tunnacliffe A, Micklem G. Expression of multiple horizontally acquired genes is a hallmark of both vertebrate and invertebrate genomes. Genome Biology. 2015;16(1):1–50. doi:10.1186/s13059-015-0607-3.
