# Supplementary figures and images for: AvP: A software package for automatic phylogenetic detection of candidate horizontal gene transfers
Source: PLoS Comput Biol. 2022 Nov 9;18(11):e1010686. doi: 10.1371/journal.pcbi.1010686 (PMC9678320; doi:10.1371/journal.pcbi.1010686)

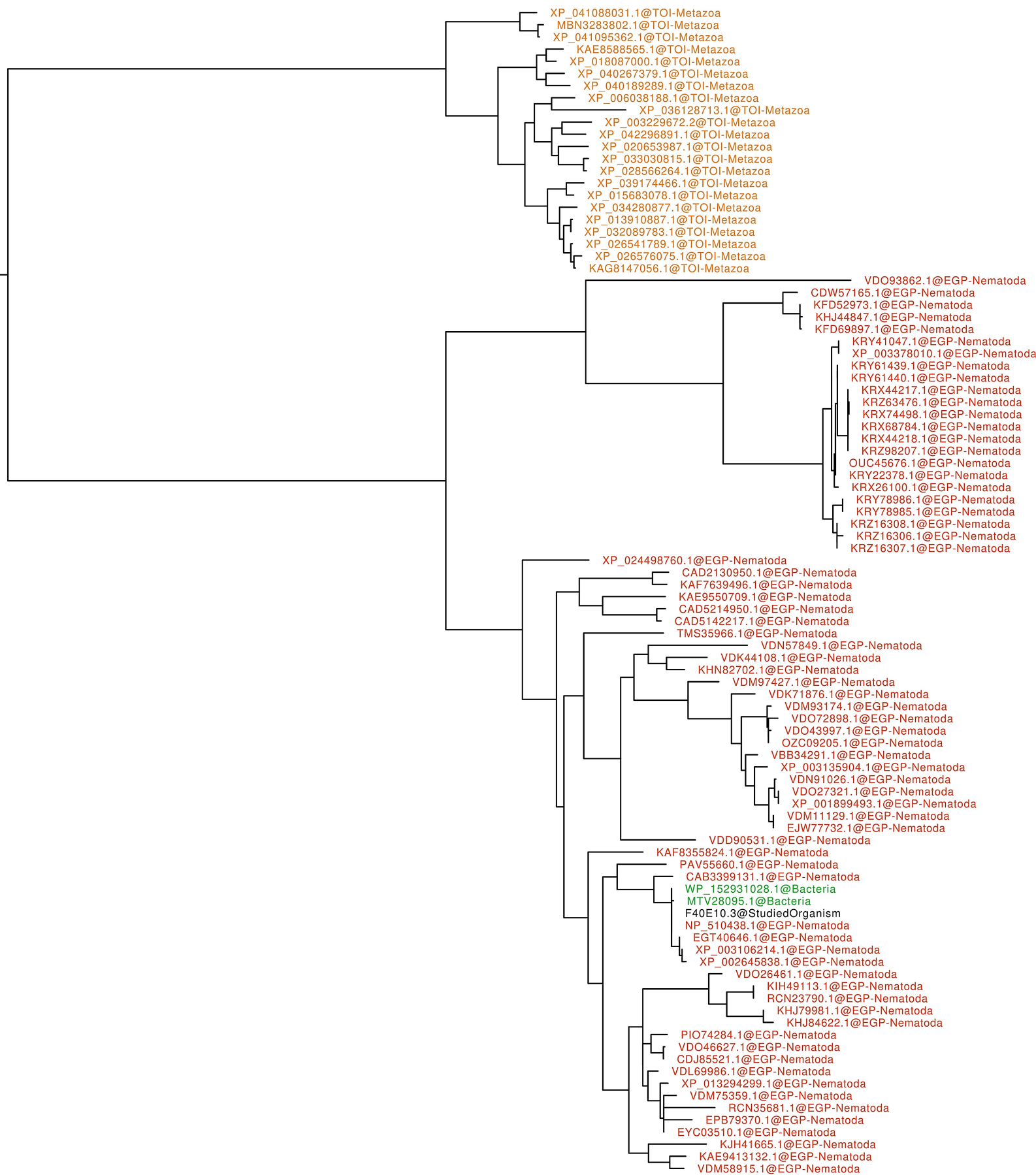

0.3

Supplement: S1 Fig — Nematoda proteins are excluded from the analysis (dark orange). Bacteria proteins are coloured green while Metazoan proteins are coloured orange. The two bacterial proteins returning the best non-metazoan hits belong to Escherichia coli and Nitriliruptoraceae bacterium and are almost identical to the protein from C. elegans indicating that they are missclasified. (PDF) [file pcbi.1010686.s002.pdf]

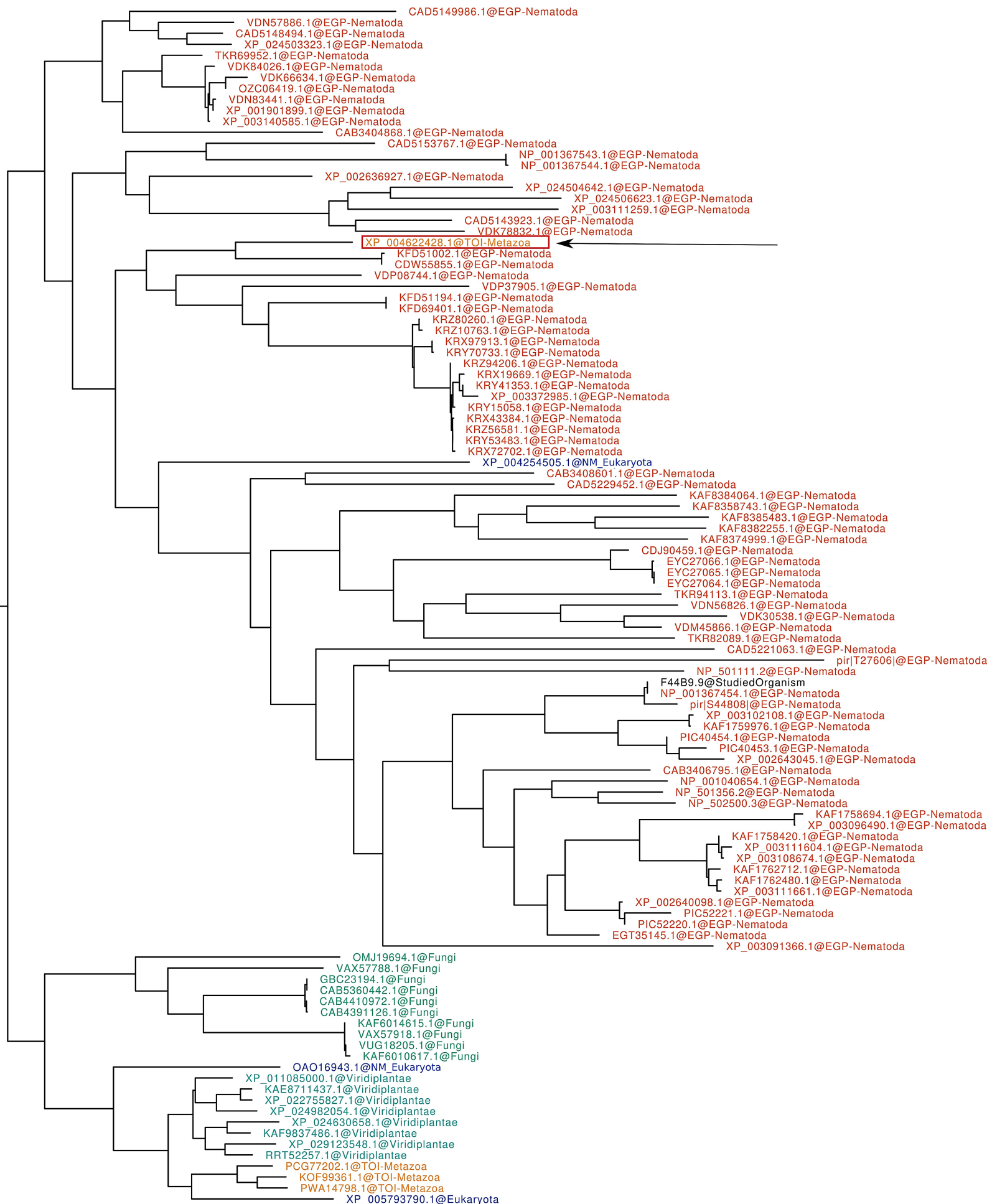

Supplement: S2 Fig — Nematoda proteins are excluded from the analysis (dark orange). Fungal proteins are coloured light green, Metazoan proteins are coloured orange, Viridiplantae proteins are coloured teal, and other non-metazoan eukaryotic proteins are coloured blue. The best Metazoan hit (excluding nematode proteins) marked with the arrow most likely belongs to a nematode from Trichuris genus. (PDF) [file pcbi.1010686.s003.pdf]
