# Supplementary material for: AvP: A software package for automatic phylogenetic detection of candidate horizontal gene transfers
Source: PLoS Comput Biol. 2022 Nov 9;18(11):e1010686. doi: 10.1371/journal.pcbi.1010686 (PMC9678320; doi:10.1371/journal.pcbi.1010686)
Supplement: Attachment — Submitted filename: response.rtf [file pcbi.1010686.s004.rtf]

Dear Dr Koutsovoulos,

The manuscript has been reviewed by two experts in the field and overall impressions of the software and manuscript were positive, although both reviewers requested greater clarity in the writing and explanation of certain points. Reviewer 2 point 2 has requested the software to detect non-protein coding genes, which is an excellent suggestion and of reat value to the field, however I am mindful that this may require extensive retooling of the software. I would like to flag this software feature as optional for the authors to address, however it must be mentioned as a limitation/future direction in the discussion of the manuscript along with any challenges that may be apparent when detecting HGT of non-coding genes. I look forward to receiving a revised version of the manuscript.

Regards,

Mark Ziemann, PhD
Dear Dr. Ziemann,
Many thanks for your evaluation of our manuscript and software. We are glad overall impressions were positive and as you will see in the point by point response to reviewers comments we have made our best to address the comments and suggestions.
We hope this revised version of the manuscript and software will be acceptable for publication in PLoS Computational Biology.

Reviewer's Responses to Questions
Comments to the Authors:
Please note here if the review is uploaded as an attachment.
Reviewer #1:
 Koutsovolous et al. present AvP, a pipeline to automatically identify horizontally transferred genes, along with a set of metrics that allow users to evaluate these predictions. By using phylogenetics at its core, AvP represents a substantial improvement compared to existing best BLAST hit-based approaches. Moreover, the metrics they develop help to address many of the issues with HGT detection (e.g. contamination in assemblies and databases). I was able to install the software from GitHub and the documentation is clear. I have no doubt that AvP will be well used and well cited by researchers interested in HGT. I struggled to find anything to complain about and, as such, I would be happy to recommend the manuscript once a few of my very minor comments (below) have been addressed/considered.
We thank reviewer 1 for the very positive and encouraging comments as well as the relevant suggestions that improve the clarity of the manuscript and software.
1. Lines 32-38: It took me a few readings of this paragraph to work out that the authors were suggesting that the existing methods that reconcile gene/species trees were not ideal because of the requirement of a species tree - could it be reworked slightly to make the message clearer? Even adding “However” before “To achieve this” would help. Note there are a few typos in here too: hundred > hundreds, compare > comparing, wth > with.
We agree this paragraph was not evident to understand and have reformulated the different sentences to make the message clearer.
2. Lines 57-59: “AI features file can be automatically generated with the Alienness webserver” - I note that this is scored out in the GitHub instructions; is this no longer true?
Due to the changes made in the format of AI features file to provide better control on selecting metrics for running AvP, the Alienness webserver AI features file is no longer compatible with AvP. However, we are working to update Alienness as soon as possible.
3. Lines 91-92: “is all not in Ingroup” > “is all species not in Ingroup”
Thanks for having spotted this missing precision that we have now corrected.
4. Lines 153-154: note that the sequenced Sciento strain of tardigrades was redescribed as H. exemplaris. I’ll let the authors decide if they'd rather use H. dujardini for consistency with previous publications or H. exemplaris for taxonomic correctness.
Thanks for pointing this update in the taxonomy and official name of the sequenced strain, we have updated the manuscript accordingly.
5. Lines 185-204: The authors only discuss the two problematic cases in their run on C. elegans, which is valuable, but what about all the other predicted HGT loci? How many were there? Were any of interest or consistent with previous work? I understand that analysing this in-depth is beyond the remit of this manuscript, but it seems odd to not mention anything other than where this had problems. A few examples of what can be discovered with the pipeline would be a valuable addition.
Indeed, we have only used C. elegans as an example to assess whether the newly introduced AHS metric was able to correct some biases associated with the Alien Index. We agree that only presenting the problematic cases can appear insufficient and in addition to the problematic casse, we have now provided a comparative analysis of the AvP results of C. elegans against those obtained using HGT index in a previously published analysis by Crisp et al., Genome Biology, 2015 (S1 File). Of the 139 HGT cases initially reported by Crisp et al., three were pseudogenes and one has been removed from Wormbase meanwhile. Thus, 135 could be compared with our AvP analysis of the C. elegans genome. Overall, of these 98 were classified as HGT by AvP, 16 were classified as complex and 21 as nont HGT. Further manual inspection of the phylogenetic trees confirmed 102 cases as HGT, 21 as Complex cases and 12 as non HGT. We did not include a report of these results in this section of the manuscript because the focus of this part was to assess the AHS score. We found that this score was able to retrieve 5 previously identified HGT cases that would have been missed by using Alien Index only. The detailed results are available in S1 File.
6. Lines 201-203: “Consequently, it seems that this new AHS metric is able to correct errors due to contamination and taxonomic assignment bias.” Perhaps I’m confused but doesn’t the AHS value, which suggests that the rodent protein is a strong HGT candidate (10356), suggests the opposite? Surely AHS is positively misleading in this case, and even more so than AI? Or are the authors suggesting that it’s the difference between AHS and AI that is indicative of a false positive?
We completely agree the way we formulated this example was misleading and, actually what we suggested here is that this was a poorly supported case based on AI resolved as a highly supported case by using the AHS score. In this case, the best metazoan hit is assigned a rodent taxonomy and this taxonomic annotation is probably wrong because a BLAST analysis revealed best hits with diverse nematodes that are rodent parasites. Consequently, the Alien Index is slightly positive because only the best non-metazoan and metazoan hits are considered and the non-metazoan one is slightly better than the (wrongly-assigned) metazoan one. In contrast, because the AHS considers the ensemble of hits the weight of the wrongly annotated metazoan sequence is compensated by the sum of non-metazoan hits and the score is highly positive and strongly suggesting an HGT event.
7. Github: it would be great if the authors could add some worked examples (e.g. the tardigrade and/or C. elegans examples in the paper) to show users how to set this up.
We have updated the GitHub documentation and added a tutorial section. 

Reviewer #2: 
I appreciate that Koutsovoulos et al. put multiple steps that are used to detect HGT into one automatic tool called “AvP”. The study is written well and the tool is friendly to users.
We thank reviewer 2 for the positive comments and we indeed tried our best to automate as much as possible the different steps in a user-friendly software.
I have four main concerns that authors might wish to address.
1. The AvP incorporated the AI values and phylogenetic robustness. Not sure how different AI cutoff will influence outputs. Of courses, there is no standard criterion. Given the complexity of the database, sometimes, the database could contain contamination so that the query protein is highly similar to the hit from contamination. A recent paper (https://pubmed.ncbi.nlm.nih.gov/35853453/) has considered anther parameter “outg_pct”, that is the percentage of species from OUTGROUP lineage in the list of the top 1,000 hits that have different taxonomic species names. Did you consider this too? If not, why or discuss it?
Yes, indeed we fully agree with reviewer 2 and one major problem to consider and tackle is database contamination and / or erroneous taxonomic annotation. Taking this problem into consideration was the main reason why we developed the AHS metric. Similarly to the outg_pct the AHS metric is not restricted to the best INGROUP and DONOR (=outgroup) hits but takes into consideration all the INGROUP and DONOR hits. Compared to the outg_pct the AHS metric proposes an additional refinement and instead of being a simple sum or percentage, this is an aggregated sum of bitscores normalized by the rank of the hits. However, we think the outg_pct metric is useful and informative and have added this calculation to the AI features file. We also have updated the program to be able to select genes for phylogenetic analyses based on any combination of the three metrics (AI, AHS, outg_pct).
2. The AvP is aiming to detect HGT based on protein-coding genes (or protein sequence), which has been done many previous studies (although they built their own pipelines). Would you please extend your tool to non-coding sequences? If you can make it, that will be much helpful, because nearly all published studies have not done this. And I think non-coding sequences could aslo be HGT too.
We fully agree that non-coding genes can also be transferred horizontally between different organisms, although their functional impact is less evident to deduce. So far, AvP was restricted to protein-coding genes and all the metrics have been calibrated to work at the protein level. However, AvP can also work at the gene level whether or not it is coding, provided that results of a homology search and associated taxonomic information are given as input. We have now added a data type option (AA or DNA, default AA: aminoacid), and we have tested the software against simulated DNA datasets. We have marked it as experimental until used in real datasets.
3. It seems that the graph neural network (or machine learning) was used to predict the HGT. if it is possible, please compare your tool with it in term of accuracy of HGT identification. If this’s hard, it would be good to discuss this aspect in your study for future direction.
We were not aware of any available software implementing neural networks or other machine learning methods to detect HGT in any kingdom of the tree of life (like AvP allows). DeepHGT (Li et al. 2020 BMC Genomics) uses deep learning to detect HGT insertion regions among prokaryotic genomes alone but not to detect HGT events themselves. Another method named GCN-HGT (Zhou et al. 2021, Science Advances) uses a graph convolutional network to predict HGT. However, so far this method has only been tested on prokaryotes and like all Machine Learning methods it needs to be trained on a big dataset of accurately validated true positive HGT cases. In that sense, we think AvP is definitely a useful tool to help rapidly construct a dataset of phylogenetically-validated HGT cases. Broader usage of AvP is expected to contribute to building a more massive dataset of trustable HGT cases that can then feed Machine Learning algorithms to train models and eventually predict HGT even more rapidly in the future directly from sequence features themselves. We agree this would be an interesting perspective and we mentioned this in the revised version of the paper.
4. If The AvP tool can also report the characteristics of HGT including the codon usage bias, dn/ds, the sequence similarity between donor and recipient, which would be better.
All the downstream analyses proposed by reviewer 2 completely make sense and would indeed constitute an interesting perspective for future developments. To include statistics on codon usage bias or dn/ds it would be necessary to retrieve the CDS corresponding to all the INGROUP and DONOR proteins. This is not straightforward as there is not always a CDS corresponding to a protein and the linkage information between CDS and proteins is not systematically provided  in protein databases.Therefore, at this stage we think it is out of the initial scope of AvP which is simply to propose a method for rapid identification of HGT cases supported by phylogenetic analysis. 
